# Key Technologies of New Type of Intravascular Ultrasound Image Processing

**DOI:** 10.3389/fsurg.2021.770106

**Published:** 2022-01-24

**Authors:** Youping Xiao

**Affiliations:** School of Information Engineering, Southwest University of Science and Technology, Mianyang, China

**Keywords:** new intravascular ultrasound, image processing, data preprocessing, three-dimensional reconstruction, cardiovascular diseases

## Abstract

Since entering the 21st century, the application of ultrasound technology has developed rapidly. Intravascular ultrasound technology has been widely used in the diagnosis and treatment of cardiovascular diseases. With the help of computer image processing technology, it can provide clinicians with more accurate diagnosis. Based on the information to improve the success rate of clinical treatment. Based on this, this article combines the development history of intravascular ultrasound technology, explores the principles of new intravascular ultrasound technology, and analyzes the application of new intravascular ultrasound technology. On this basis, the preprocessing of intravascular ultrasound image data is discussed, involving the acquisition of intravascular ultrasound image data and image analysis. On this basis, explore the combined application of new intravascular ultrasound technology and other imaging examination methods, such as X-rays to use three-dimensional image technology to reconstruct new intravascular ultrasound image sequences, and provide doctors with clearer morphology and properties of tube wall lesions. In order to make a more accurate diagnosis of the lesion, a more detailed and accurate treatment plan can be given, which has extremely high clinical application value.

## Introduction

Intravascular ultrasound (Intravascular ultrasound, also referred to as IVUS) is a new technology that combines non-invasive ultrasound technology and invasive cardiac catheterization technology to diagnose cardiovascular diseases. The clinical application of intravascular ultrasound technology is to insert a miniaturized vascular ultrasound probe into the patient's cardiovascular cavity through cardiac catheter technology, detect and display the shape and image of the cardiovascular cross-section on the electronic imaging system, and analyze the patient's heart and blood Image of blood flow inside the tube (1, 2). Compared with other inspection methods, the application of intravascular ultrasound technology not only allows doctors to more clearly grasp the specific shape of the patient's vascular cavity, but also intuitively understand the structure of the blood vessel wall, which can more accurately judge the degree of disease of the blood vessel wall Based on this characteristic, intravascular ultrasound is also known as the new “gold standard” for vascular examination. Although the earliest development of intravascular ultrasound can be traced back to the 1970s and 1980s, the technology of intravascular ultrasound has developed rapidly in recent years, especially the image quality has been greatly improved (3–5). The ultrasound frequency is 20–40 MHZ. The directional accuracy can reach up to 20 μm, and its imaging accuracy is much higher than other imaging inspection methods, and it has a wide range of clinical value (6, 7). At present, intravascular ultrasound technology is often used in two aspects: one is for the detection of early symptoms of coronary atherosclerosis, and the other is the formulation of interventional clinical treatment plans to evaluate the effect of treatment. Based on the ultrasound signal, accurate representations of normal, fibrotic, calcific and vulnerable plaques (plaques with necrotic core) are configured by the IVUS processing system (8). The three-dimensional morphology of the patient's blood vessel can be used to quantitatively analyze the vascular diseased plaque, so as to provide more accurate information for the doctor's interventional treatment plan, which can greatly improve the accuracy of clinical medical diagnosis and treatment (9, 10). Shengnan Liu et al. (11) developed and validated an optimized framework for accurate automated detection and quantification of calcified plaque in coronary atherosclerosis as seen by IVUS, which may play a role in image-guided coronary interventions. Therefore, it is necessary to rely on computer image processing technology to process intravascular ultrasound imaging, which can automatically extract and reconstruct the blood vessel wall.

## Analysis of Intravascular Ultrasound Image Processing Technology

### Development of Intravascular Ultrasound

**Figure 1:**
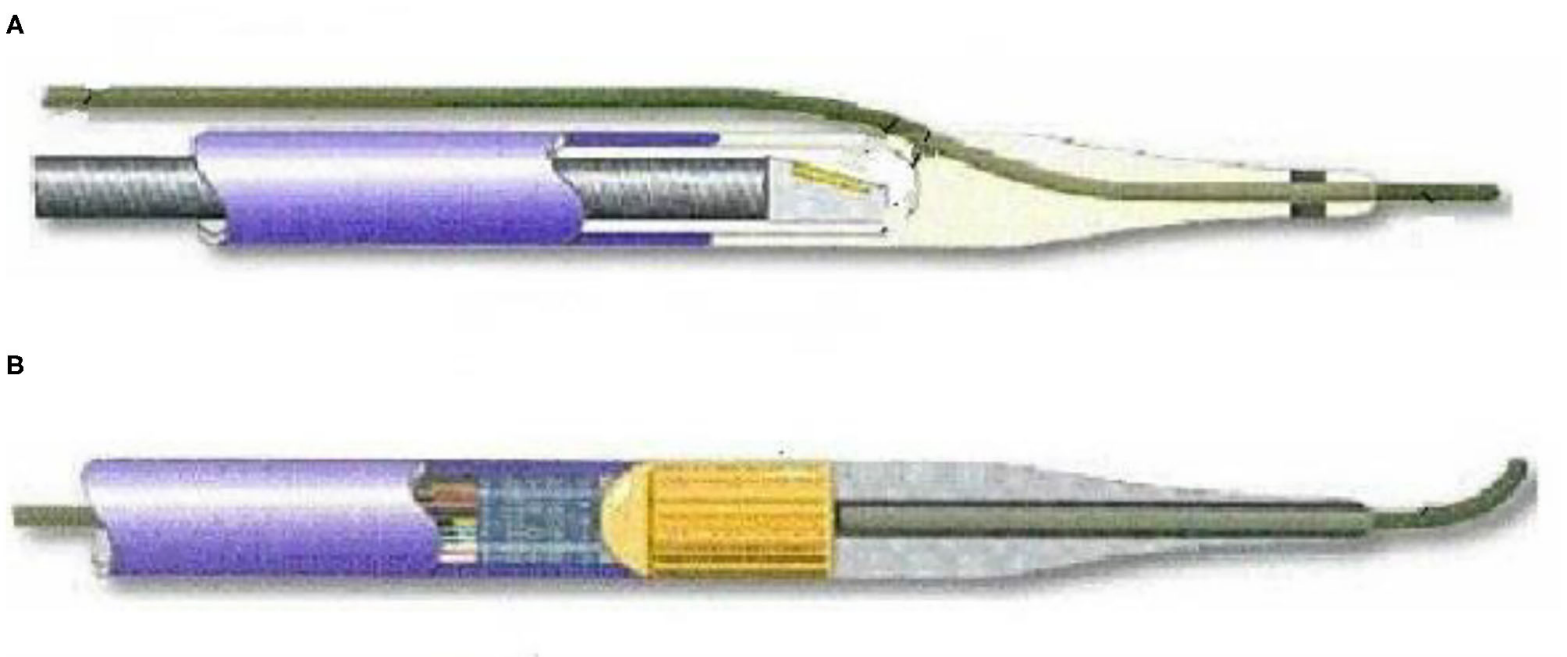
Rotary and phased array ultrasound probes.

The history of the development of intravascular ultrasound can be traced back to an ultrasound catheter technology for intracardiac ultrasound measurement in 1956. It was found that the ultrasound catheter was able to obtain soft tissue echoes in a laboratory model, and it was found that the ultrasound catheter was in a medical examination. Application possibilities. In 1971, Bom invented a 32-crystal phased array ultrasound probe, which can display real-time images of the patient's heart section. In 1974, Olson invented an ultrasound probe that passes through the patient's esophagus, which can check the blood flow of the patient's aorta. In the 1980s, the emergence of ultrasound radiation processing technology further promoted the development of clinical applications of medical ultrasound (12, 13). With the clinical application of the miniaturization of ultrasound probes, the mechanical rotation type and phased array type of intravascular ultrasound technology have replaced the traditional manual rotation type, and have greatly improved the safety and reliability of the application of ultrasound technology. Rotary and 64-crystal phased array ultrasound probes are commonly used clinically, as shown in Figure 1.

As above, where A is a rotary mechanical ultrasonic probe, which mainly maintains the motor to rotate at a constant speed through the rotating shaft, so that the transducer is guided to rotate, sends and receives signals, and draws images. B is a phased array ultrasound probe. Through the electronic phased array system, it enters into the internal arrangement of ultrasonic sensor groups. The vibration generates ultrasonic waves and transmits them in the form of beams with the aid of the medium. According to the difference in reflection and absorption of different ultrasonic waves by human blood vessels, the feedback is different. Acoustic signal and present the image on the computer as a basis for doctors to judge the nature of vascular disease (14, 15).

### Doppler Effect

In 1842, when the Austrian mathematician and physicist Christian Andreas Doppler observed the movement of stars, he discovered a special physical phenomenon, that is, moving stars will undergo blue shift and red shift. When the stars move toward the earth, the frequency will become higher and the light color will be increased. A blue shift phenomenon will occur, and when the stars are far away from the earth, the frequency will become lower, and the light color will have a red shift phenomenon, which is also known as the “Doppler effect.” According to the principle of Doppler effect, intravascular ultrasound technology is used to estimate the blood flow velocity in the blood vessel (16, 17). Assuming that the ultrasound transmitting source of intravascular ultrasound technology is stationary, its ultrasound frequency is f_0_, and the mass propagation velocity of ultrasound is c, and the velocity is v, then the ultrasound frequency is expressed as (c+v) f_0_/c. When the ultrasonic wave reaches the moving object, the ultrasonic wave reflects back to the transmitting source and is received. Here the frequency of the moving object is expressed as (c+v) f0/(c-v), and the Doppler frequency shift f_d_ is expressed as $\frac{2\text{v}}{\text{c}-\text{v}}f_{0}$. For example, the direction of the object movement, there is an angle ϑ with the direction of the ultrasonic signal, and the Doppler frequency shift formula of the object motion component in the direction of the ultrasonic signal is calculated as:


(1) $$\begin{array}{r} f_{d}=\frac{2\text{V}cos\theta }{c}f_{0} \end{array}$$

The propagation speed of ultrasound signals on human tissues is about 1,540 m/s, and the ultrasound transmission frequency is usually 3–10 MHz. The formula for calculating blood flow in human blood vessels using the Doppler frequency shift formula is:


(2) $$\begin{array}{r} \text{V}\left(\text{cm}/\text{s}\right)=\frac{77\left(\text{cm}/\text{ms}\right)\times {\text{f}}_{0}\left(\text{KHz}\right)}{{\text{f}}_{0}\left(\text{MHz}\right)\times \text{cos}\theta } \end{array}$$

In addition, note that the Doppler included angle will have some influence on the accuracy of the final blood flow velocity. If the included angle ϑ is too large, the measurement error will be greater, and if the included angle is too small, it will also cause too much refracted energy. It is too small to measure blood flow velocity. Therefore, in clinical applications, the Doppler angle ϑ is generally set to the interval of 45–60°.

### Principles of New Intravascular Ultrasound Technology

**Figure 2:**
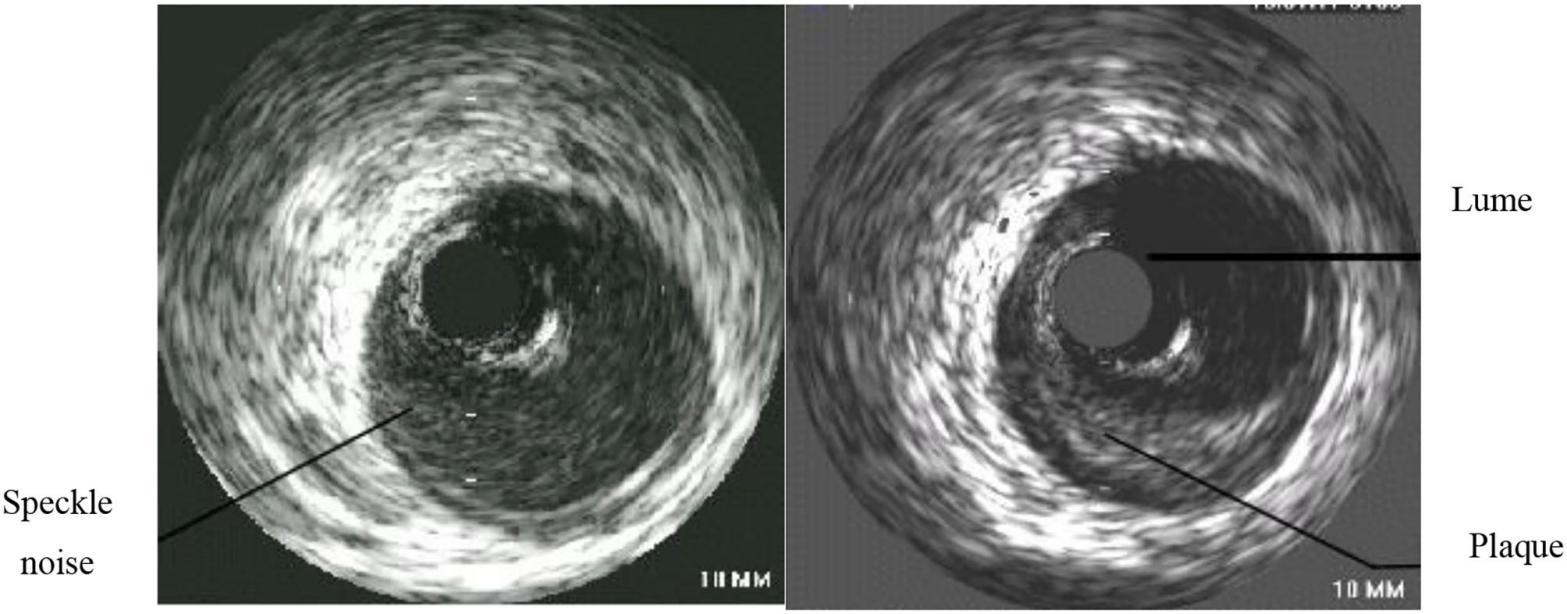
Comparison of normal coronary artery and diseased coronary artery.

After the new type of intravascular ultrasound catheter enters the patient's blood vessel, through computer technology, the two-dimensional cross-sectional image of the blood vessel at the target location can be displayed on the ultrasound instrument for the doctor to see if it is diseased. The doctor constantly adjusts the depth of field according to the observation position, that is, the size of the cross-sectional image, and can view the full picture of the blood vessel, which is used as the most reasonable image for disease analysis (18, 19). Through intravascular ultrasound technology, the patient's arterial lumen inner diameter and shape can be presented. The image is shown in Figure 2. The left side is the normal coronary artery structure and wall thickness shape. The lumen on the image is circular and encircling. The lumen has a layered structure, and the blood flow distribution in the lumen also presents an approximately circular shape, and the visualization is a continuously changing hypoechoic or anechoic zone. On the right is the image of the lesioned coronary artery structure and wall thickness. The atherosclerotic plate in the coronary artery structure is the majority, and the image is abnormal echo area, accompanied by the increase of the lumen thickness and the reduction of the lumen area.

**Figure 3:**
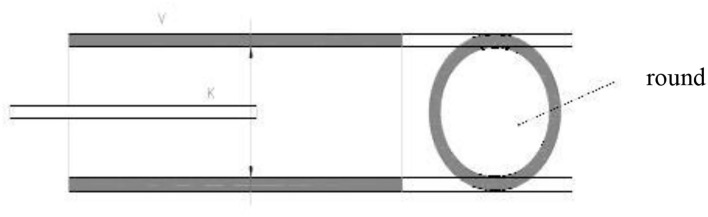
The position of the ultrasound catheter in the center of the blood vessel.

**Figure 4:**
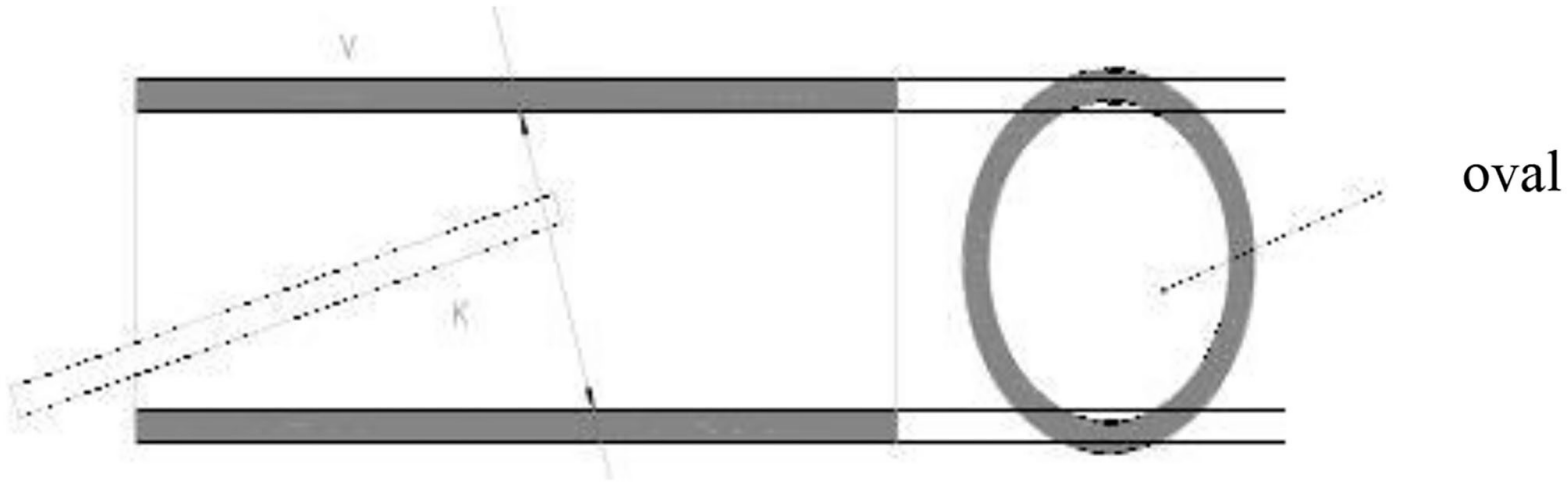
Ultrasound catheter in the non-central position of the blood vessel.

Note that in order to obtain more accurate and reliable images, the operation of the intravascular ultrasound instrument should be standardized, and the gain condition should be adjusted according to the inspection needs to minimize the interference of image artifacts and retain the most important information that can be used as the basis for the inspection. During the continuous pullback acquisition of coronary artery IVUS studies, the spatial consistency between consecutive frames is corrupted by the vessel wall displacement, periodical change in vascular dimensions and the catheter movement relative to the vessel wall caused by pulsatile blood and cardiac motion. It is known as motion artifact, which is decomposed into two spatial components, longitudinal (or axial) motion and in-plane motion (20, 21). The former relates to the forward or backward longitudinal displacement of the catheter along the vessel axis. The latter relates to the relative translation and rotation between the ultrasonic transducer and the lumen. It produces the misalignment of the vessel structures from vessel reconstruction are hindered for non-diastolic phases since the vascular cross-sections are not equally located in space along the catheter pullback trajectory. During the operation of intravascular ultrasound, the ultrasound is affected by the systolic and diastolic activity of the patient's heart, and under electromagnetic interference, the image presents artifacts, which affects the accuracy of the doctor's condition analysis (22, 23). When operating an intravascular ultrasound instrument, place the tip of the catheter in the center of the lumen and keep it parallel to the long axis of the blood vessel. However, this is the most ideal situation, and it is often impossible to achieve this state in actual operation. As shown in Figure 3, when the tip of the intravascular ultrasound catheter is parallel to the long axis of the blood vessel, its cross-section is circular. As shown in Figure 4, when the tip of the intravascular ultrasound catheter is in a non-central position, it is not parallel to the long axis of the blood vessel, and the cross-sectional shape of the blood vessel is also shown as an ellipse.

**Figure 5:**
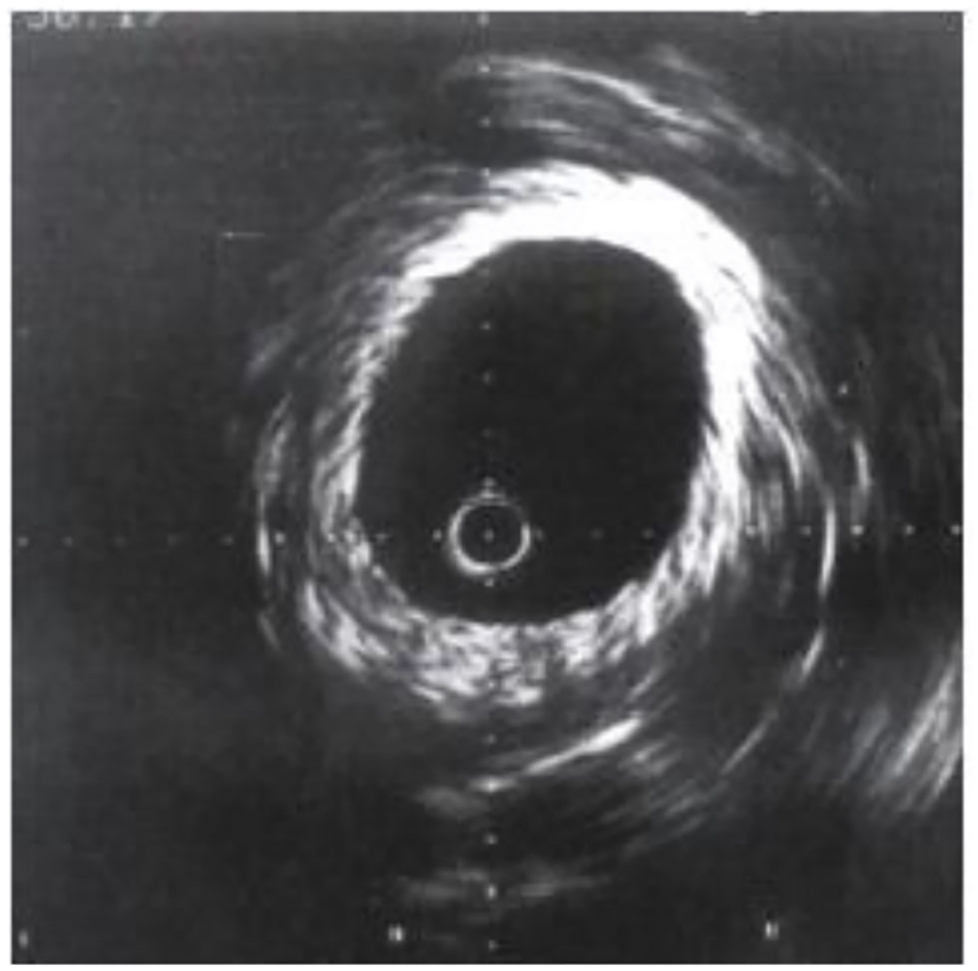
Ultrasound image of non-central position.

As shown in Figure 5, the intravascular ultrasound instrument catheter is in a non-central position of the patient's blood vessel. The ultrasound image displayed clearly shows that the cross-sectional shape of the blood vessel is elliptical, and the imaged blood vessel wall will have obvious changes in strength and weakness echo.

**Figure 6:**
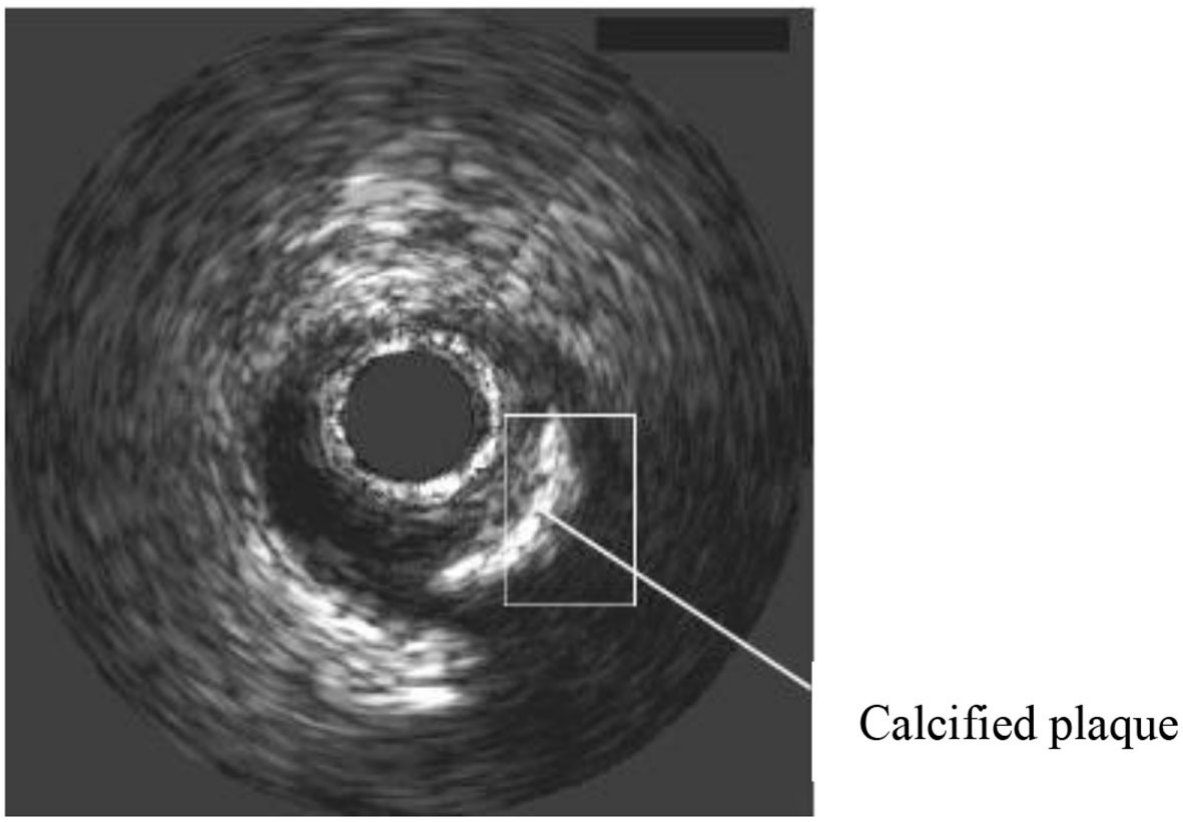
Ultrasound vascular acoustic shadow.

During the operation of the intravascular ultrasound instrument, if there is human tissue with high acoustic impedance, the sound beam will be perpendicular to the reflection interface, and multiple reflection imaging will appear, and the returned part of the sound beam will be reflected on the interface again and repeated many times. Due to the high acoustic impedance generated by the tissue of the calcified plaque, the ultrasonic sound beam cannot really penetrate the calcium, and the sound beam will be reflected by the calcified plaque boundary, which will form an acoustic shadow. As shown in Figure 6, intravascular calcification. The tissue morphology behind the plaque cannot be visualized.

In the process of IVUS examination, various factors may cause artifacts, mainly including motion artifact mentioned above, non-uniform rotation distortion (NURD) and ringdown artifact the artifact affects the image quality, interfering with identification on the plaque properties. The IVUS system includes an imaging console, a pull-back system, and an integrated catheter. in the conventional mechanical rotation type of integrated catheter, when a flexible shaft driven by a motor passes through a narrow space, uneven rotation occurs, which is caused by a change in the torsional force due to the varying friction force. This phenomenon may cause image distortion, also known as NURD. Several methods have been proposed to solve NURD, mainly focus on majorization of the piezoelectric motor to make it meet the requirements of the internal driver of the IVUS catheter (24). The ringdown artifact is caused by the ringdown effect when activate the ultrasonic piezoelectric crystal, which manifests a white image around the ultrasonic catheter. Ringdown interference is avoided by modifying transducers and applying filters. Usually transducers modification and filters application are perfprmed to avoid Ringdown interference (25). Therefore, when operating an intravascular ultrasound instrument, doctors must consider the effects of artifacts from multiple aspects as much as possible, as well as the measurement errors of the ultrasound system, in order to obtain more accurate and reliable image results.

## Preprocessing of Intravascular Ultrasound Image Data

Due to the complex structure of human blood vessels, echoes of different intensities will occur even at the same tissue location. In addition, calcified plaques will also affect the echo status, and the image morphology is also more complicated, and the automatic edge extraction technology often cannot really improve Ultrasound image accuracy. Therefore, the active contour model is used for image edge extraction to provide doctors with clear and reliable examination image information.

### Acquisition of Intravascular Ultrasound Image Data

The preprocessing of intravascular ultrasound image data mainly includes two aspects: first, to remove the artifacts of the intravascular ultrasound catheter; second, to eliminate the marker points of the intravascular ultrasound image. The characteristics of the intravascular ultrasound catheter will cause a dead zone in the center of the patient's blood vessel image, and the surrounding area will be surrounded by halo artifacts, which will affect the initial contour of the vascular intima. However, when the region of interest at the edge of the blood vessel intima is near the intima, the halo artifact in the center of the image can be recorded as an invalid region to eliminate interference. Therefore, the doctor will directly locate the center of the circle at the center of the ultrasound image, and when the other radius exceeds the circle mark defined by the ultrasound catheter size, it will be marked as an invalid area (26). At the same time, in order to save the running time of the algorithm as much as possible, the area outside the boundary of the outer membrane is generally marked as an invalid area, and finally a ring-shaped effective area is obtained.

### Active Contour Model Image Edge Extraction

Contour extraction is considered to be an autonomous, bottom-up processing process, and the bottom processing results will directly affect the top processing. Given that, in 1987, Kass (27) proposed an active contour model the model could deal with the information in the low-level process obtained from the high-level process to get rid of the constraint of strict stratification. The model can integrate image data, initial values, target contour features, knowledge constraints, etc. into the extraction of image features, and perform initialization processing to autonomously converge to a state of minimal energy. This algorithm has high applicability, not only suitable for different types of biological tissues, but also for different personalities, and is in line with the needs of medical clinical image processing. Therefore, this article analyzes the edge extraction method of the active contour model image.

The essence of the active contour model is the energy function, which is minimized to find the target contour of the image. It fully considers the internal energy of the active contour, the image force, and the energy influence generated by the external limiting force, so that the target contour maintains smoothness, Continuity. The deformable curve of the active contour model is v(s) = {x(s), y(s)}, where s takes a value on [0,1], and its total energy function formula is:


(3) $$\begin{array}{r} E_{snake}=\underset{0}{\overset{1}{\int }}\left[E_{int}\left(v\left(s\right)\right)+E_{image}\left(v\left(s\right)\right)+E_{con}\left(v\left(s\right)\right)\right]ds \end{array}$$

In the formula, E_*int*_ represents the internal energy condition of the curve determined by the derivative, E_*image*_ represents the energy derived from the image data, and E_*com*_ represents the external control force energy.

The internal energy E_*int*_ is expressed as E_*int*_ = α(*s*)|*v*′(*s*)|^2^+β(*s*)|*v*”(*s*)|^2^ by the formula, where α(*s*) represents the control profile elastic weighting coefficient, β(*s*) represents the stiffness weighting coefficient, *v*′(*s*) represents the first derivative, and *v*”(*s*) represents the second derivative.

The formula of the image energy E_*image*_ is expressed as $E_{image}=-\gamma \left(s\right){\left|\nabla I\left(x,y\right)\right|}^{2}$, which γ(*s*) represents the weight coefficient and ∇*I*(*x, y*) represents the grayscale gradient of the image.

The external control force energy E_*com*_ is not a necessary item of the energy function, and is generally defined according to actual usage requirements. For intravascular ultrasound image processing, based on Kass's active contour model, Williams (28) proposed a fast active contour model for contour extraction, based on the model algorithm, the contour is updated by calculating the local minimum energy of the contour points. Hence, the fast active contour model has the advantage of fast calculation speed and meets the needs of medical clinical image contrast.

In the fast active contour model, discrete data is used to present digital images, and the discrete form is expressed by the formula:


(4) $$\begin{array}{r} E=\overset{N_{e}}{\underset{i=1}{\sum }}\left[\alpha \left(i\right)E_{cont}\left(i\right)+\beta \left(i\right)E_{curv}\left(i\right)+\gamma \left(i\right)E_{mage}\left(i\right)\right] \end{array}$$

In the formula, Nc represents the total number of control points, represents internal energy, and represents image energy. There is no definition of external control energy. The discrete approximation of is expressed by the following formula:

In the formula, *N*_*c*_ represents the total number of control points, α(*i*)*E*_*cont*_(*i*)+β(*i*)*E*_*curv*_(*i*) represents internal energy, and γ(*i*)*E*_*mage*_(*i*) represents image energy. There is no external control energy defined here. The discrete approximation of *E*_*comt*_(*i*) is expressed by the following formula:


(5) $$\begin{array}{r} E_{cont}\left(i\right)=\bar{d_{c}}-|V_{i}-V_{i-1}| \end{array}$$

In the formula, $\bar{d_{c}}$ represents the average distance of each control point, and |*V*_*i*_−*V*_*i*−1_| refers to the distance between the control point and the previous control point. When the distance between the two control points is close to the average distance, the energy value is small, and each control point on the image contour also presents an even distribution. The discrete approximate formula of curvature *E*_*curv*_(*i*) is expressed as:


(6) $$\begin{array}{r} E_{curv}\left(i\right)={\left|V_{i-1}-2V_{i}+V_{i+1}\right|}^{2} \end{array}$$

In the formula, *i*+1 refers to the latter control point, *E*_*comt*_(*i*) and *E*_*curv*_(*i*) are divided by the maximum value for normalization.

The image energy *E*_*mage*_(*i*) is processed by the image gray gradient to obtain the normalized result, as follows:


(7) $$\begin{array}{r} E_{image}\left(i\right)=\frac{g_{min}-g_{i}}{g_{max}-g_{min}} \end{array}$$

In the formula, *g*_*i*_ represents the current control point gradient value, while *g*_*max*_ and *g*_*min*_ represent the maximum and minimum gradients in the field. Based on the above formula, it can be known that the higher the value of *g*_*i*_, the smaller the value of *E*_*mage*_(*i*), and can make the control point move to the edge of the larger gradient value.

The image edge extraction through the fast active contour model can not only improve the running speed of image processing, but the use of *E*_*comt*_ calculation formulas can make the distribution of the control points of the image contour line even, and also tend to be smooth.

### Analysis of Intravascular Ultrasound Images

When using the new intravascular ultrasound technology for vascular examination, after the image edge is extracted through the fast active contour model, some plaque tissues in the image need to be automatically identified, classified, and evaluated, and on this basis, the intravascular cavity. The progression of the plaque is predicted to develop an appropriate treatment plan. Specifically, it is divided into two types based on plaque recognition in intravascular ultrasound images:

First, based on the image analysis method, the plaque tissue is calibrated mainly through the analysis of the texture characteristics of the gray image, combined with the theoretical knowledge of the relevant luminal plaque characteristics. However, the changes in the characteristics of human tissue plaques are extremely complex, and the imaging of ultrasound images also has certain limitations, which affects the accuracy of the determination of vascular luminal plaques, and cannot really distinguish the properties of plaques, such as fibrous and lipid. Sexuality, etc., need to cooperate with other examination techniques to assist clinical examinations.

Second, clinically, the signals of new intravascular ultrasound catheters can generally be classified to identify different plaque properties in the vascular lumen. The new type of intravascular ultrasound radio frequency signal is used to reconstruct the gray image and analyze the texture of the blood vessel structure. However, it should be noted that not all types of intravascular ultrasound instruments allow the collection of original radio frequency signals, thereby affecting the identification and classification of the properties of luminal plaques.

## The Combined Application of new Intravascular Ultrasound Technology and Other Imaging Methods

### New Intravascular Ultrasound Technology

**Figure 7:**
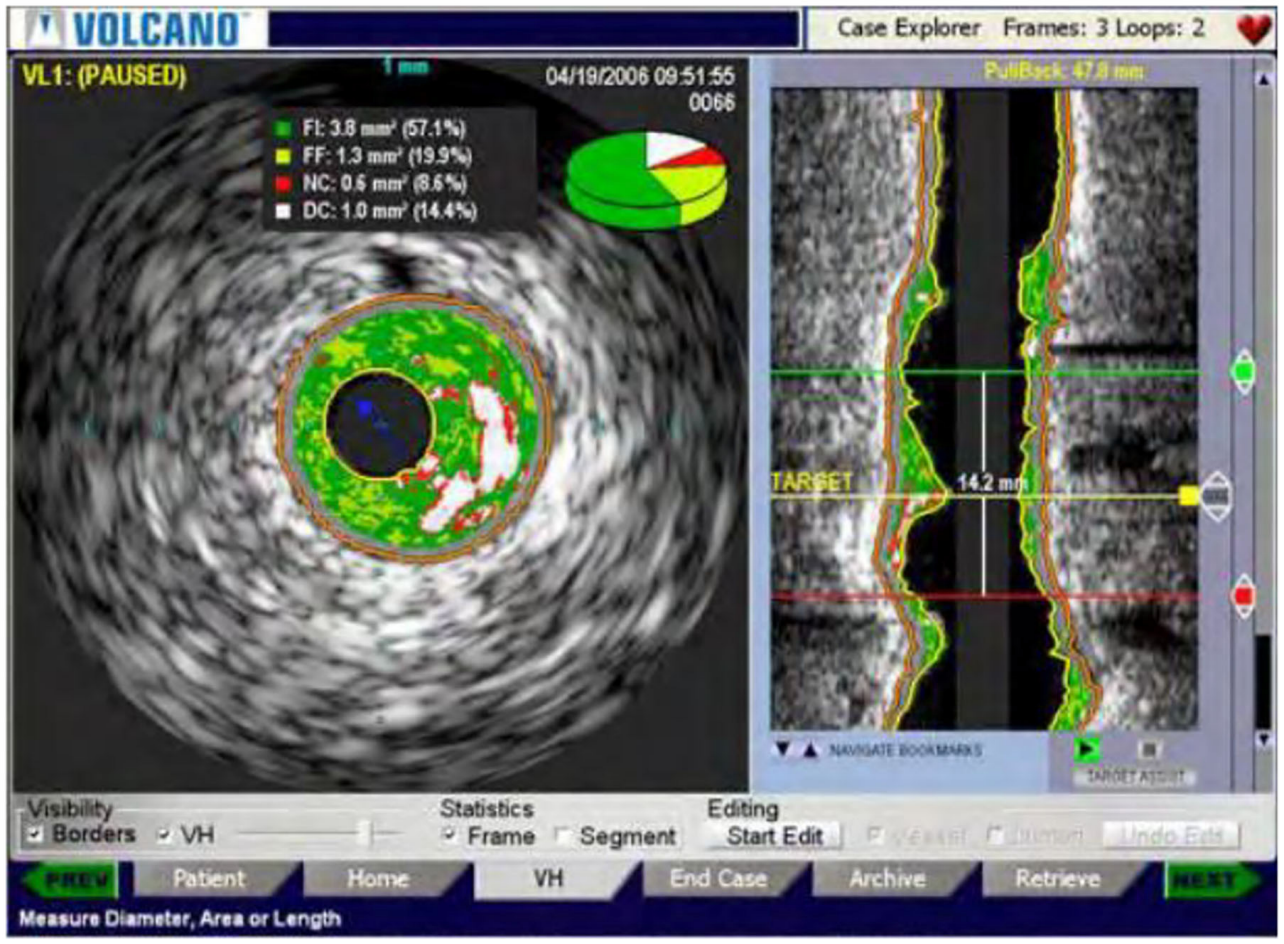
VH-IVUS image of a new type of intravascular ultrasound.

In order to obtain more accurately the distribution of plaques on the inner wall of the patient's blood vessel, a new type of intravascular ultrasound technology was added to the original foundation of the virtual tissue imaging technology, that is, VH technology, as well as vascular elasticity. Among them, the virtual tissue imaging technology is a brand-new technology developed by Volcano in the United States (29). The operating principle is to measure the back-radiation integral of the ultrasonic echo signal, and establish a connection with the database that has been built before, and it can partition the plaques with different colors. differentiate. As shown in Figure 7, Volcano's VH-IVUS technology can collect RF data through ECG gating. If the patient's R wave amplitude is too low, clear images cannot be obtained. The vascular elastography technology is a technology that presents the biomechanical characteristics of the vascular wall and plaque. The IVUSE technology based on image processing does not need to capture the original radio frequency signal, so as to process the gray-scale image, and calculate the horizontality of the vascular plaque. The normal strain and shear strain of the cross-section, drawing a two-dimensional elastic map, can more comprehensively and clearly reflect the elastic properties of the plaque in the lumen.

### Combined With X-Ray Angiography Technology

**Figure 8:**
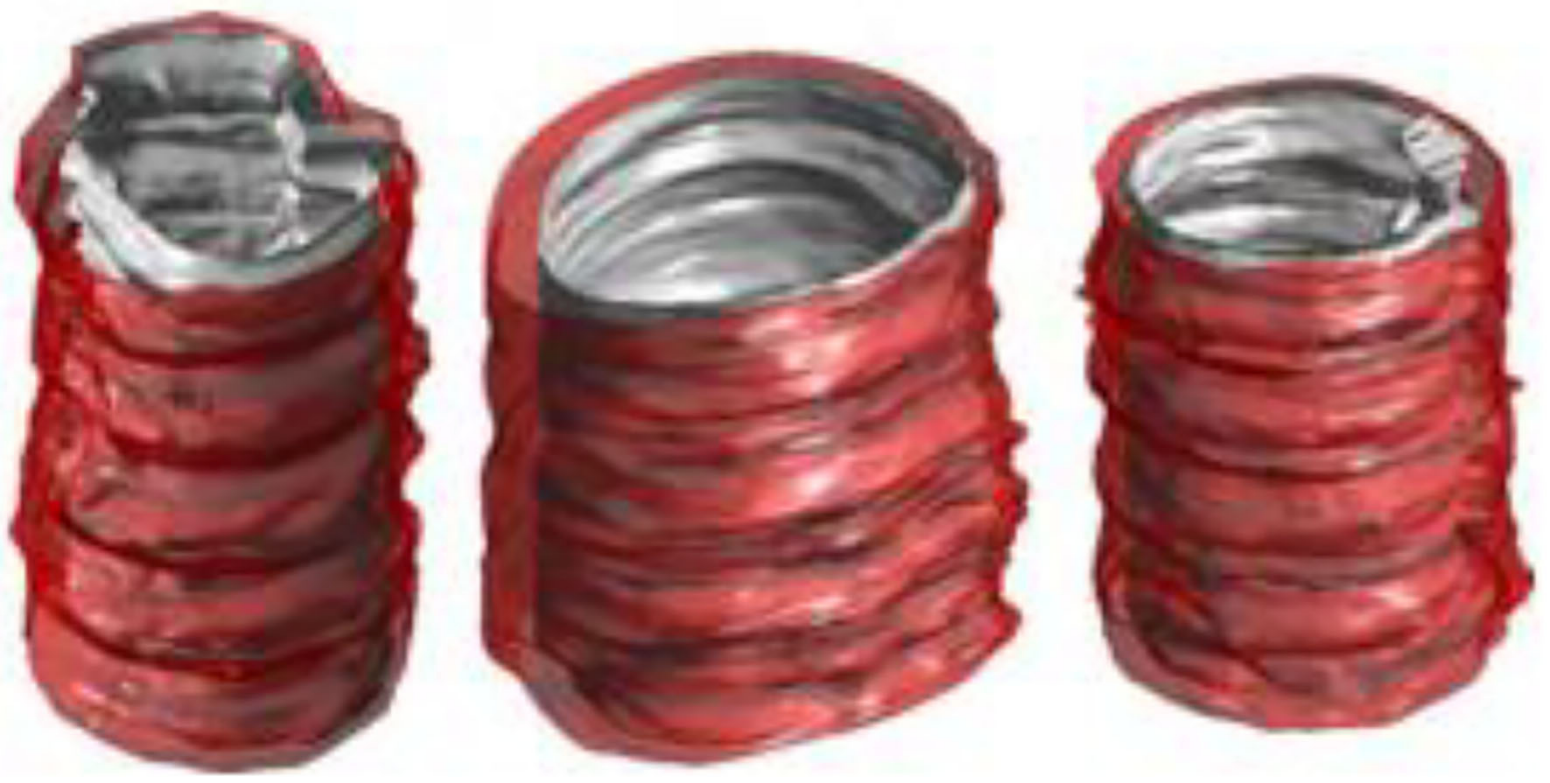
New type of intravascular ultrasound image sequence blood vessel reconstruction results.

The three-dimensional reconstruction of blood vessels in traditional clinical examination applications is mainly to superimpose a series of intravascular ultrasound images according to the time sequence of phenomena to form three-dimensional blood vessel segments, as shown in the Figure 8. In the process of three-dimensional reconstruction of blood vessels, the bending characteristics of blood vessels are generally not considered. Sliding during intravascular ultrasound withdrawal operation is likely to cause loss and distortion of information, or artifacts, which affect the accuracy and reliability of the image.

**Figure 9:**
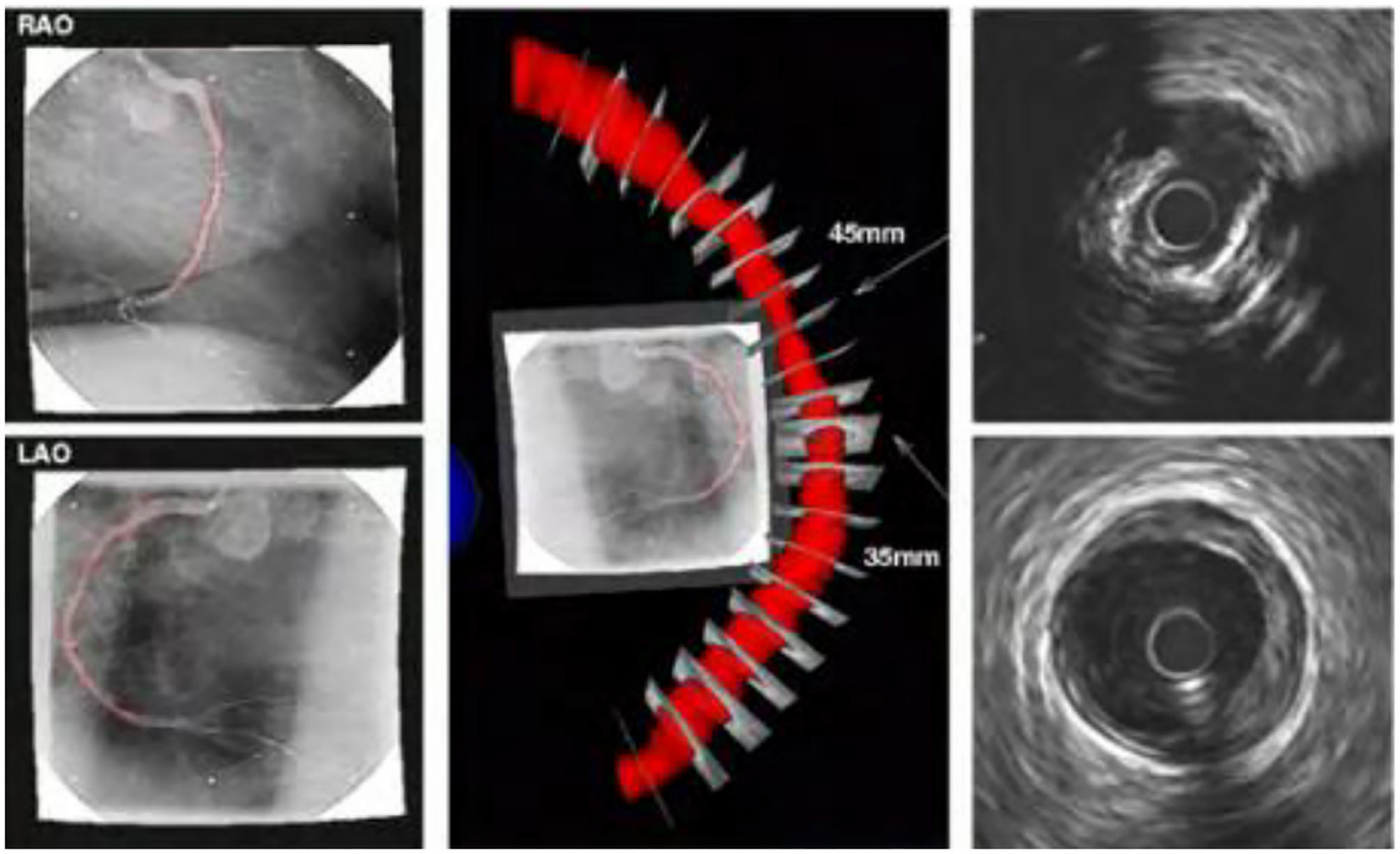
Reconstruction results of new type of intravascular ultrasound combined with X-ray angiography.

The combination of intravascular ultrasound technology and X-ray angiography can simultaneously display the structure and shape of the vascular cavity, and reconstruct the vascular structure through computer three-dimensional technology, as shown in the Figure 9. In this process, the synchronous collection of information can cover multiple cardiac cycles of intra-coronary ultrasound image sequences, thereby realizing dynamic three-dimensional reconstruction of the targeted vascular segment, and reproducing the true shape of the coronary arteries during the cycle.

The new intravascular ultrasound technology and X-ray angiography have some complementarities in clinical application. X-ray angiography can show the contour after the contrast agent is filled, but cannot obtain the morphological structure of the vessel wall and plaque. The new intravascular ultrasound technology can visualize the cross-section of the blood vessel, but cannot locate the spatial position of the plaque. Combining these two technologies to achieve complementary advantages can not only achieve three-dimensional reconstruction of blood vessels, but also overcome the shortcomings of a single technology.

### Combining IV-OCT Image Technology

**Figure 10:**
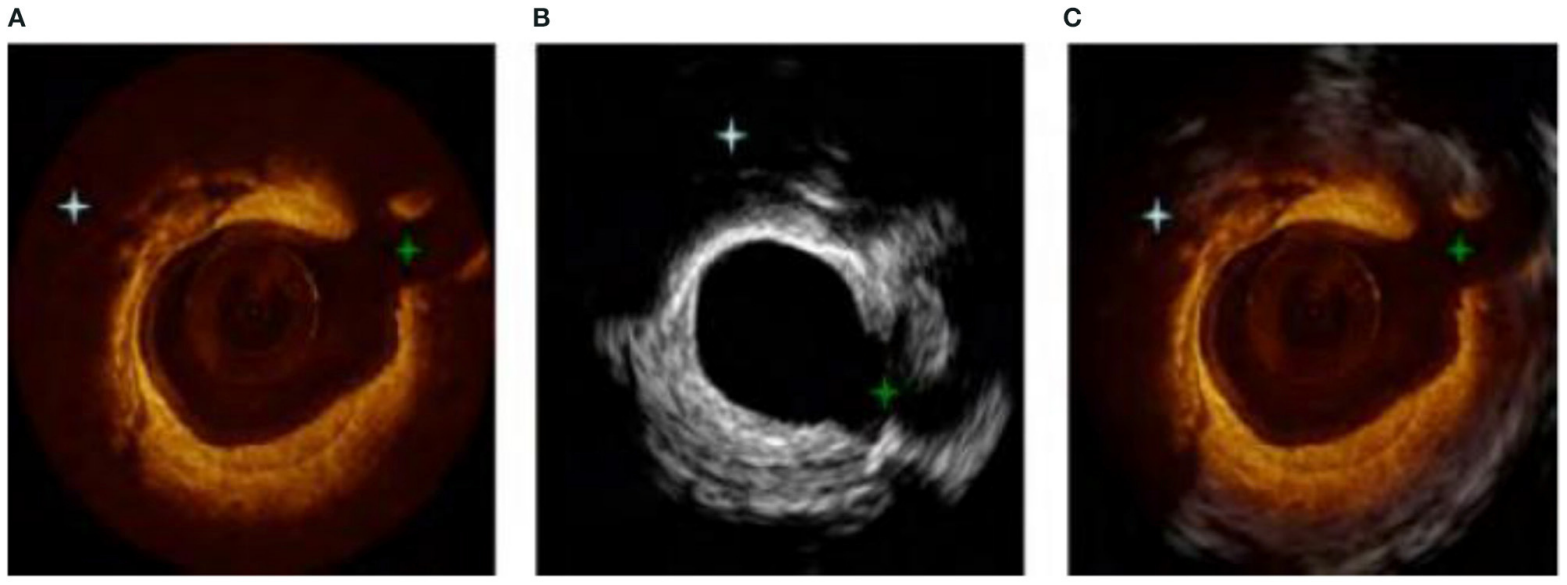
**(A–C)** Combination image of new intravascular ultrasound technology and IV-OCT.

The IV-OCT imaging technology has some similarities with the operation principle of the new intravascular ultrasound technology. The energy beam is used to scan the internal structure of the patient's vascular cavity in an all-round way, and to obtain a cross-sectional image contrast of the blood vessel. However, the two contrast techniques also have some complementarity (30, 31). The new intravascular ultrasound technology can detect the depth of blood vessels, but cannot obtain higher-resolution images, and the structure of some small tissues is not accurate. The IV-OCT imaging technology can just make up for this defect. Its axial and lateral resolution is high, but it uses infrared light source rays, which have limited penetration into human tissues. Therefore, the combination of the new intravascular ultrasound technology and IV-OCT imaging technology can integrate the advantages of high resolution and high penetration, so as to fully describe the structure of the blood vessel wall, as shown in Figure 10. Figure 10A is an IV-OCT image contrast, Figure 10B is a new type of intravascular ultrasound image, and Figure 10C is a combination of the two technologies. Previous studies have shown that OCT-guided percutaneous coronary intervention (PCI) using a specific reference segment external elastic lamina-based stent optimisation strategy was safe and resulted in similar minimum stent area to that of IVUS-guided PCI (32), IVUS-guided PCI also potentially improving acute and long-term patient outcomes compared to angiography-guided PCI (33). However, it should be noted that these two inspection methods may not be performed at the same time, so the catheter retraction path is not the same, resulting in errors in the image sequence.

In addition, due to the influence of the patient's heart movement, the image data collection points of each cardiac cycle will also be different, which will directly affect the structure of the blood vessel and increase the difficulty of the inspection and registration.

### Combined With CT Angiography Technology

CT angiography is a non-invasive vascular imaging technology, which is often used clinically on the anatomical structures of patients. Therefore, combining the new intravascular ultrasound technology with CT angiography technology can use the new intravascular ultrasound technology to reconstruct the vascular lumen structure in three dimensions, and use CT angiography technology to reconstruct the centerline of the blood vessel, which is similar to the catheter retraction path. Image. In the actual application process, the patient's vessel lumen axis may not coincide with the retraction path of the new type of intravascular ultrasound. Therefore, the doctor should take into account the errors caused by this situation when performing three-dimensional reconstruction of the vascular structure, and use. The evaluation algorithm also takes the error into consideration when calculating.

## Conclusion

In summary, the new type of intravascular ultrasound instrument applied to the clinic has high operational feasibility and auxiliary diagnostic value. In order to obtain more accurate image information, a variety of computer image processing techniques are often used for image processing. Among them, the use of active contour model image edge extraction has higher operational advantages and has the value of wide promotion and application. The signals collected by the new intravascular ultrasound technology can objectively analyze human tissues, vascular lumen morphology, and plaque properties through advanced data analysis of ultrasound signals. However, image sequence processing based on the new type of intravascular ultrasound also has some limitations. It can be combined with other inspection methods, such as X-ray angiography, IV-OCT image technology, CT angiography, etc., through complementary advantages, can provide doctors with comprehensive accuracy the basis of screening diagnosis, and can improve the effect of surgical treatment, has a high application and promotion prospects.

## Author Contributions

The author confirms being the sole contributor of this work and has approved it for publication.

## Conflict of Interest

The author declares that the research was conducted in the absence of any commercial or financial relationships that could be construed as a potential conflict of interest.

## Publisher's Note

All claims expressed in this article are solely those of the authors and do not necessarily represent those of their affiliated organizations, or those of the publisher, the editors and the reviewers. Any product that may be evaluated in this article, or claim that may be made by its manufacturer, is not guaranteed or endorsed by the publisher.
